# EphrinB3 blocks EphB3 dependence receptor functions to prevent cell death following traumatic brain injury

**DOI:** 10.1038/cddis.2014.165

**Published:** 2014-05-08

**Authors:** M H Theus, J Ricard, S J Glass, L G Travieso, D J Liebl

**Affiliations:** 1 Department of Biomedical Sciences and Pathobiology, Virginia-Maryland Regional College of Veterinary Medicine, 215 Duck Pond Drive, Blacksburg, VA 24061, USA; 2 Miami Project to Cure Paralysis, Department of Neurological Surgery, University of Miami Miller School of Medicine, 1095 NW 14th Terrace, R-48, Miami, FL 33136, USA

**Keywords:** ephrins, Eph receptors, controlled cortical impact (CCI) injury, traumatic brain injury (TBI)

## Abstract

Eph receptor tyrosine kinases and their membrane-bound ligands, ephrins, have a variety of roles in the developing and adult central nervous system that require direct cell–cell interactions; including regulating axon path finding, cell proliferation, migration and synaptic plasticity. Recently, we identified a novel pro-survival role for ephrins in the adult subventricular zone, where ephrinB3 blocks Eph-mediated cell death during adult neurogenesis. Here, we examined whether EphB3 mediates cell death in the adult forebrain following traumatic brain injury and whether ephrinB3 infusion could limit this effect. We show that EphB3 co-labels with microtubule-associated protein 2-positive neurons in the adult cortex and is closely associated with ephrinB3 ligand, which is reduced following controlled cortical impact (CCI) injury. In the complete absence of EphB3 (EphB3^−/−^), we observed reduced terminal deoxynucleotidyl transferase-dUTP nick end labeling (TUNEL), and functional improvements in motor deficits after CCI injury as compared with wild-type and ephrinB3^−/−^ mice. We also demonstrated that EphB3 exhibits dependence receptor characteristics as it is cleaved by caspases and induces cell death, which is not observed in the presence of ephrinB3. Following trauma, infusion of pre-clustered ephrinB3-Fc molecules (eB3-Fc) into the contralateral ventricle reduced cortical infarct volume and TUNEL staining in the cortex, dentate gyrus and CA3 hippocampus of wild-type and ephrinB3^−/−^ mice, but not EphB3^−/−^ mice. Similarly, application of eB3-Fc improved motor functions after CCI injury. We conclude that EphB3 mediates cell death in the adult cortex through a novel dependence receptor-mediated cell death mechanism in the injured adult cortex and is attenuated following ephrinB3 stimulation.

Traumatic brain injury (TBI) is a multifaceted condition initiated by mechanical tissue disruption and induction of a secondary phase of injury that triggers both necrotic- and apoptotic-related events leading to neuronal loss, axonal destruction and demyelination.^1, 2, 3, 4, 5, 6^ These events are accompanied by long-term cognitive and neurological deficits in humans^7^ as well as in rodent models of experimental brain injury.^8, 9, 10^ A number of studies have demonstrated that TBI-induced neuronal damage is a critical component of the secondary injury, and apoptotic mechanisms account for the majority of cell death.^11, 12, 13, 14^ Neuroprotective strategies aimed at preventing apoptosis, cellular damage and the neuropathological sequelae of TBI have largely failed to translate to clinical treatment. Thus, additional understanding of the molecular mechanisms that regulate apoptotic-mediated neuronal loss following central nervous system (CNS) trauma is warranted.

Ephrins and Eph receptors have traditionally been shown to have important roles in the development of the CNS.^15, 16, 17, 18^ Although we know a great deal about their influence in development, a growing number of studies support their role in the injured adult CNS.^19, 20^ In particular, temporal expression differences are observed on neurons and glial cells following injury,^21, 22, 23, 24, 25^ which in turn function to mediate injury-induced axon sprouting, cellular remodeling and glial scar formation.^21, 22, 26, 27, 28^ Recently, we described a novel ‘dependence receptor' function for EphA4 receptors, where these receptors initiate cellular apoptosis in the absence of their ligand(s).^29^ In another study, EphB3 displayed effects that strongly supported its affiliation to the dependence receptors family.^30^ Dependence receptors form a family of receptors that can transduce both positive and negative signals depending on ligand interaction. During normal tissue homeostasis, dependence receptors interact with their cognate ligand to transduce positive cellular changes, such as survival, differentiation and/or migration; however, in conditions where ligand–-receptor interactions are reduced, these receptors initiate or amplify programmed cell death. Therefore, dependence receptors create a cellular state of dependence for their ligand. Activation or elimination of this unique phenomenon is thought to have a key role in embryonic development, neurodegenerative diseases and cancer progression. To date, dependent receptor mechanisms have not been appreciated or adequately examined in the context of CNS injury.

Here, we demonstrate that EphB3 mediates cell death in the acute phase of controlled cortical impact (CCI) injury, a murine model of TBI. In the absence of EphB3, we observed increased cell survival and improvements in motor recovery as compared with deficiencies in ephrinB3 or wild-type mice. This supports the unique role of Eph-mediated cell death, where loss of the receptor, but not of the ligand, is protective. Furthermore, like EphA4 and many other dependence receptors, EphB3 undergoes cleavage by caspase(s) to initiate cell death mechanism. Stimulation studies using soluble clustered ephrinB3-Fc (eB3-Fc) support this role, where stimulating Eph signaling reverses CCI injury-induced phenotypes. These findings provide evidence for a dependence receptor function of EphB3 in the adult brain following trauma, and represent a unique target for neuroprotective strategies.

## Results

### EphB3 and ephrinB3 expression in the adult murine cortex and hippocampus

**Figure 1:**
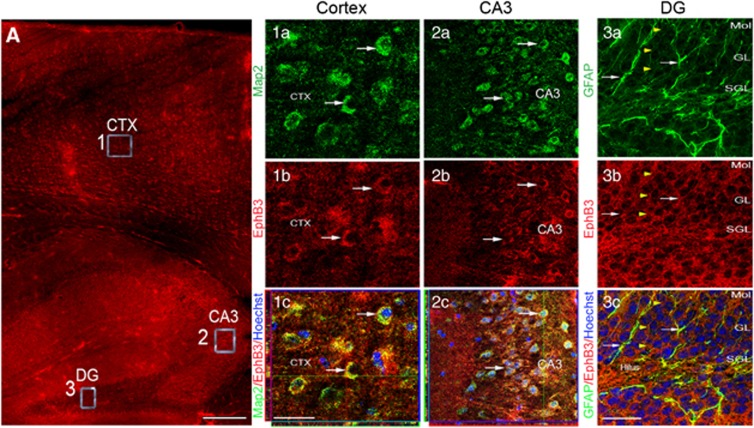
EphB3 expression in the adult murine forebrain. EphB3 (**A**) is expressed by MAP-2-positive cells in the naive adult cortex (inset 1; 1a–c) and CA3 regions (inset 2; 2a–c) as well as throughout multiple layers of the dentate gyrus (inset 3; 3a–c). EphB3 is expressed by GFAP-positive cells in the DG (3a–c). Scale bar=500 μm in image A; 20 *μ*m in E-H; 50 *μ*m in I-L

**Figure 2:**
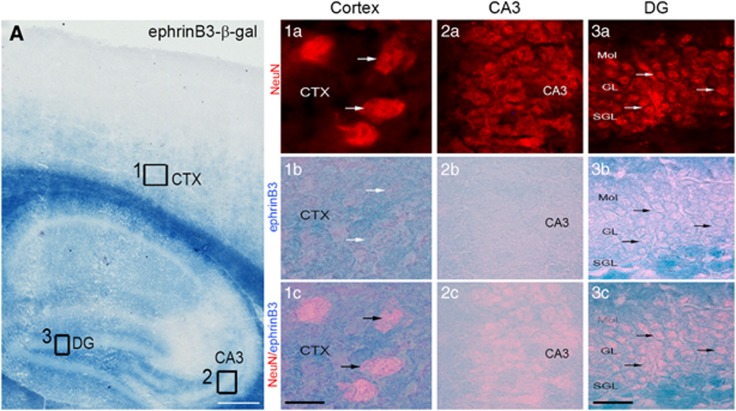
EphrinB3 expression in the adult murine forebrain. Low magnification of a sagittal brain section from ephrinB3^lacZ^ mouse (**A**). EphrinB3 (X-gal staining) is present in the corpus callosum (CC), CA3 and cortical layers IV-VI (CTX). In the cortex (inset 1), CA3 (inset 2) and dentate gyrus (inset 3) NeuN-positive cells (1a; 2a; 3a) are adjacent to ephrinB3 staining (1b and c; 2b and c). (1a–3a) NeuN-positive neurons magnified from image A, within the cortex (1a), CA3 (2a) and dentate gyrus (3a). Scale bar=500 *μ*m in image A; 20 *μ*m in 1a-1c; 50 *μ*m in 2a–3c

**Figure 3:**
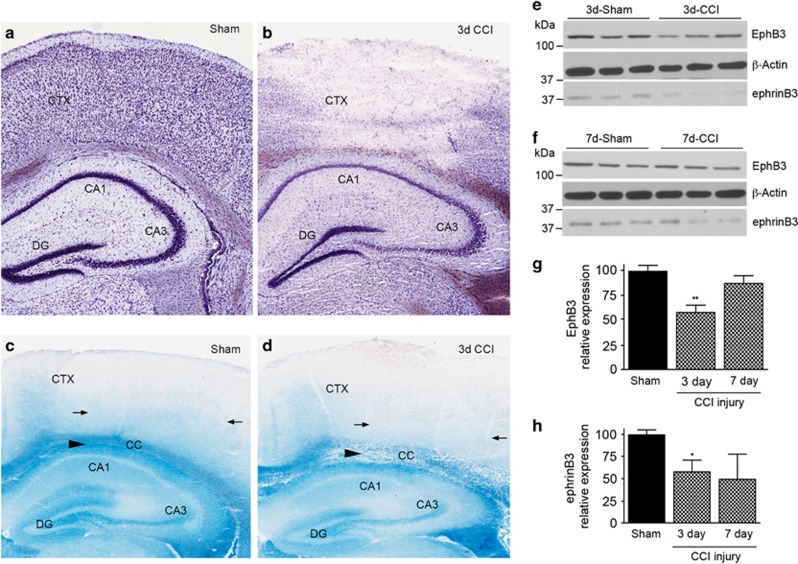
EphB3 and ephrinB3 protein levels are reduced in the cortex post CCI injury. Low-magnification images of Nissl-stained coronal sections at 3 days post sham (**a**) and CCI injury (**b**) showing selective cortical tissue loss with sparing of hippocampal structures. (**c** and **d**) X-gal staining in ephrinB3^lacZ^ mice after sham and CCI injury, respectively, demonstrates reduced staining in the cortex (CTX) and corpus callosum (C). (**e** and **f**) Western blot analysis for EphB3 and ephrinB3 expression in the cortex at 3 days (**e**) and 7 days (**f**) post injury. Significant reductions in EphB3 expression were observed at 3 days, as measured by densitometry normalized to *β*-actin control levels (**g**), whereas ephrinB3 expression was reduced, significantly, at 3 days and non-significantly at 7 days compared with sham-injured levels (**h**). **P<0.01; *P<0.05 compared with sham injury. *n*=5 per group and time point

To evaluate the role of EphB3-ephrinB3 signaling following CCI injury, we first determined their cellular localization in the adult cortex and hippocampus, as these areas are selectively damaged following moderate cortical injury. Using immunofluorescence labeling and confocal image analysis, we found that EphB3 was expressed by microtubule-associated protein 2-positive neurons in the cortex (Figures 1; 1a–c) and in the CA3 region of the adult hippocampus (Figures 1; 2a–c). We also found EphB3 expression throughout the dentate gyrus layers (yellow arrowheads) and on GFAP-positive cells (Figures 1; 3a–c). To examine ephrinB3 expression, we took advantage of a transgenic knock-in mouse where *β*-galactosidase (ephrinB3-*β*-gal) replaces the cytoplasmic domain of ephrinB3 allowing for selective labeling. Dense *β*-galactosidase expression (through X-gal staining) was observed throughout the brain, including the striatum, septum and corpus callosum (CC; Figure 2), and weaker staining within the cortical layers overlying the CC (Figures 2; 1a–c) as well as in the dentate gyrus pyramidal layers (Figures 2; 3a–c); however, in the cortex and CA3 hippocampus, staining was seen adjacent to NeuN-positive neuron cell bodies rather than within them (Figures 2; 2a–c). *β*-Galactosidase expression was observed in regions that correlate with axon and dendritic growth in the dentate granule cells and CA1 pyramidal neurons, which supports our previous studies where ephrinB3 mRNA expression was observed in the DGC and CA1 cell layers^16, 18^ and synaptic membranes.^18^ Together, these studies support a similar regional expression pattern between ephrinB3 and EphB3 in neurons that reside in both the cortex and hippocampus.

Histological assessment using Nissl staining on brain sections from CCI-injured mice showed cellular loss in the cortex 3 days after injury (Figures 3a and b). Coincident with neuronal cell loss, ephrinB3 expression visualized by X-gal staining was reduced in the cortex and CC at 3 days after CCI injury (Figures 3c and d). Western blot analysis showed a significant difference in the levels of ephrinB3 and EphB3 in the cortex at 3 and 7 days post CCI injury (Figures 3e–h). In particular, both ephrinB3 and EphB3 levels were reduced in the cortex at 3 days post CCI injury, whereas only ephrinB3 remained attenuated at 7 days. In whole hippocampal tissues, there was no significant change in the levels of EphB3 at 3 and 7 days post CCI injury. However, ephrinB3 was significantly reduced by 42% at 3 days and restored to sham levels by 7 days post injury (not shown). Together, these findings demonstrate that CCI injury leads to an acute attenuation in ephrinB3 and EphB3 expression, which could result from cell loss and/or reduced protein levels.

### Cell death and motor deficits are attenuated in EphB3^−^/^−^ mice following CCI injury

**Figure 4:**
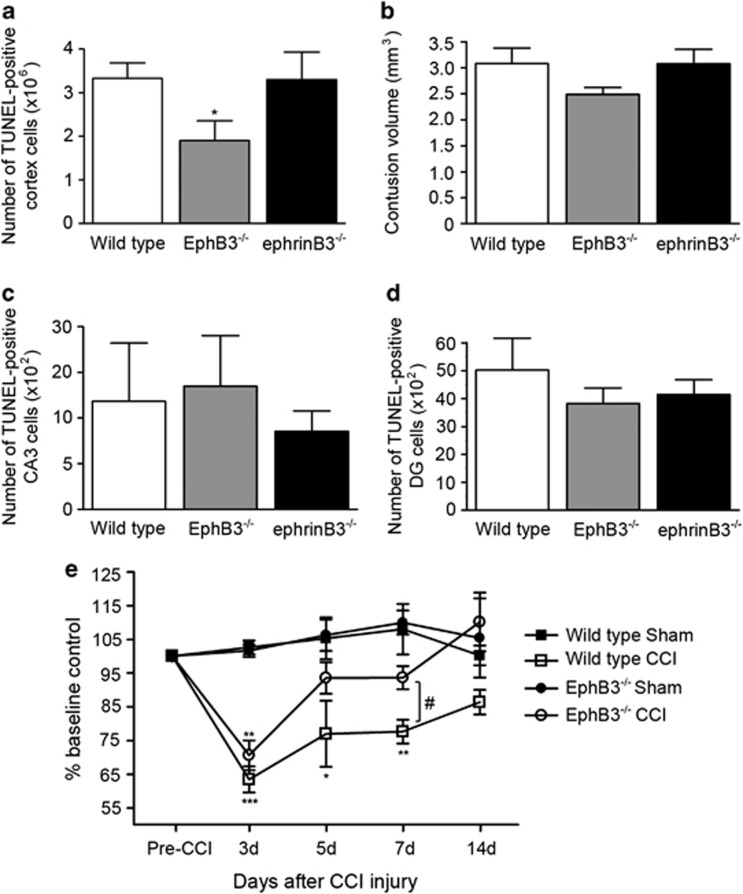
Cell death and motor deficits are attenuated in EphB3^−/−^ mice following CCI injury. Cell death was quantified in the cortex, CA3 and dentate gyrus 3 days after CCI injury in wild-type, ephrinB3^−/−^ and EphB3^−/−^ mice. (**a**) The total number of TUNEL-positive cells in the cortex was significantly reduced in EphB3^−/−^ compared with wild-type and ephrinB3^−/−^ mice; however, no significant difference in contusion volume was observed between wild-type, ephrinB3^−/−^ and EphB3^−/−^ mice (**b**). In contrast, no significant difference in the number of TUNEL-positive cells was found in the CA3 (**c**) and dentate gyrus (**d**) between wild-type, ephrinB3^−/−^- and EphB3^−/−^-injured mice. (**e**) Motor deficits following sham and CCI injury using RotaRod assessment at 3, 5, 7 and 14 days post injury in wild-type and EphB3^−/−^ mice. Compared with pre-training baseline control, wild-type mice had significant motor deficits at 3, 5 and 7 days post CCI injury (*n*=7) compared with sham-injured (*n*=5) control mice. Although CCI-injured EphB3^−/−^ mice (*n*=7) showed significant motor deficits at 3 days, functional improvement was observed at 5, 7 and 14 days compared with sham-injured controls (*n*=5), with a significant improvement over wild-type injured mice at 7 days. ***P<0.001; **P<0.01; *P<0.05 CCI compared with sham. ^#^*P*<0.05 EphB3^−/−^ CCI compared with wild-type CCI injured

To examine whether ephrinB3-EphB3 signaling has a role in CNS damage after CCI injury, cell death was analyzed by counting the number of terminal deoxynucleotidyl transferase-dUTP nick end labeling (TUNEL)-positive cells in the cortex, dentate gyrus and CA3 hippocampal regions of wild-type, ephrinB3^−/−^ and EphB3^−/−^ mice at 3 days following CCI injury. EphrinB3^−/−^ nor EphB3^−/−^ mice show no signs of phenotypic abnormalities in cortical cell numbers or cortical development. Sham-injured animals did not display any TUNEL labeling in the cortex; however, there was significant labeling in the injured cortex of wild-type animals (333 200±35 130 cells, *n*=6), which was similar to that of ephrinB3^−/−^ mice (330 200±63 070 cells, *n*=5; Figure 4a). Interestingly, there was a significant decrease in the number of TUNEL-positive cells in EphB3^−/−^ mice (190 500±44 280 cells, *n*=5) compared with wild type at 3 days following CCI injury. We also observed a non-significant decrease in contusion volume in EphB3^−/−^ compared with wild-type and ephrinB3^−/−^ mice (Figure 4b). The total number of TUNEL-positive cells was not significantly different in the CA3 (Figure 4c) or dentate gyrus (Figure 4d) regions of the hippocampus following CCI injury. To determine whether reduced cell death in the cortex of EphB3^−/−^ mice correlated with functional differences, RotaRod behavioral analysis was performed on wild-type and EphB3^−/−^ mice at 3, 5, 7 and 14 days after sham or CCI injury. We observed a significant attenuation in motor behavior of all CCI-injured mice at 3 days post injury, but only wild-type CCI-injured mice showed significant reductions from sham mice at days 5 and 7 post CCI injury (Figure 4e). At 7 days post CCI injury, EphB3^−/−^ CCI-injured mice (93.7±3.4% of baseline, *n*=7) showed significantly better motor behavior scores as compared with CCI-injured wild-type mice (77.7±3.5% of baseline, *n*=7), suggesting that EphB3 has a deleterious role following TBI that may likely occur through its ability to promote cell death.

### EphrinB3 infusion reduces cell death and motor dysfunction following CCI injury

**Figure 5:**
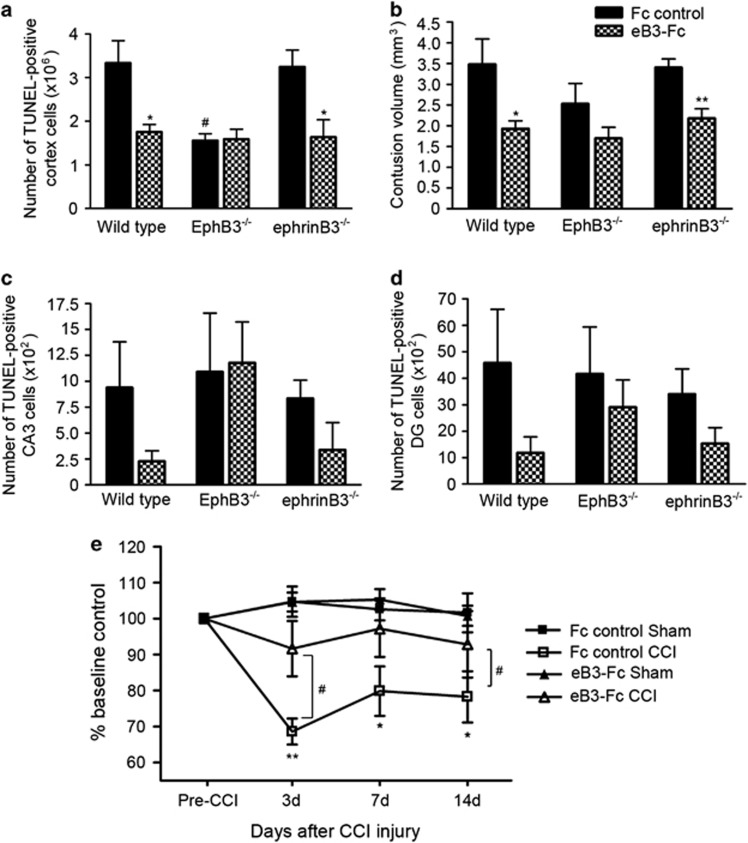
EphrinB3 infusion reduces cell death and motor dysfunction following CCI injury. Cell death was quantified in the cortex, CA3 and dentate gyrus 3 days after CCI injury in wild-type, ephrinB3^−/−^ and EphB3^−/−^ mice following infusion of either pre-clustered ephrinB3-Fc (eB3-Fc) or Fc molecules in the contralateral ventricles. (**a**) Cell death in the cortex was significantly reduced following eB3-Fc infusion in wild-type and ephrinB3^−/−^ mice compared with Fc control infusions, whereas EphB3^−/−^ mice showed reduced cell death with both eB3-Fc and Fc controls. (**b**) A significant reduction in the contusion volume (mm^3^) following eB3-Fc infusion was observed in wild-type and ephrinB3^−/−^ mice compared with Fc infusions. (**c** and **d**) There was non-significant decrease cell death in the CA3 and dentate gyrus following eB3-Fc infusions in wild-type and ephrinB3^−/−^ mice. (**e**) Motor deficits using RotaRod analysis in wild-type sham- and CCI-injured mice following infusion with either eB3-Fc or Fc molecules. Although there were significant motor deficits in CCI-injured mice receiving Fc-control, no differences were found in motor function between sham- and CCI-injured mice receiving eB3-Fc infusions. (**a**–**d**) *P<0.05 compared with corresponding Fc-control; ^#^*P*<0.05 compared with wild-type Fc controls. (**e**) **P<0.01 compared with Fc control sham-injured mice; ^#^*P*<0.05 between eB3-Fc infused wild-type and EphB3^−/−^ mice

We had previously shown that in the absence of ephrinB3 or in the presence of overexpressed Eph receptors, induction of cell death occurs.^29, 30^ Our current findings support a role for EphB3 as a potential pro-death dependence receptor following TBI. In the CCI-injured cortex, this is supported by reduced expression of ephrinB3 at 3 and 7 days post CCI injury (Figure 2), which coincides with tissue damage and functional deficits. However, it is unclear whether loss of ephrinB3 alone is sufficient to induce dependence receptor-mediated cell death. To better demonstrate the pro-survival effects of ephrinB3, we infused it directly into the brain in an attempt to block EphB3-mediated cell death. Thus, we infused soluble pre-clustered ephrinB3-Fc (1.7 *μ*g/day; eB3-Fc) molecules into the contralateral ventricles of wild-type, ephrinB3^−/−^ and EphB3^−/−^ mice for 3 days following CCI injury. We quantified contusion volume and cell death in the cortex, dentate gyrus and hippocampal CA3. We found that eB3-Fc infusion significantly reduced the number of TUNEL-positive cells in the cortex of wild-type mice (Fc control: 333 400±51 530, *n*=5 *versus* eB3-Fc: 175 200±17 630, *n*=5) and ephrinB3^−/−^ mice (Fc control: 324 800±38 400, *n*=5 *versus* eB3-Fc: 164 100±39 220, *n*=5; Figure 5a) at 3 days following CCI injury. In addition, reduced cell death observed in the cortex following eB3-Fc infusion correlated with a decrease in contusion volume in wild-type and ephrinB3^−/−^ mice (Figure 5b). In the absence of EphB3, reduced cell death was observed in the cortex but stimulation using soluble ephrinB3 ligand had no effect compared with wild-type mice (Figure 5a). A similar profile was observed in the analysis of the contusion volume between wild-type, EphB3^−/−^ and ephrinB3^−/−^ mice (Figure 5b). In the hippocampus, there was a trend toward reduced cell death following eB3-Fc infusion in the CA3 hippocampus (Figure 5c) and dentate gyrus (Figure 5d) in wild-type and ephrinB3^−/−^ mice. Little to no difference was observed in EphB3^−/−^ mice. Together, these data support EphB3-mediated neuronal and possibly non-neuronal cell death and its potential role in regulating dependence receptor-like activities in the cortex following TBI.

To evaluate whether the pro-survival effects of ephrinB3 could lead to functional improvements, we infused pre-clustered eB3-Fc or Fc molecules into sham and CCI-injured wild-type mice and assessed motor function using RotaRod behavioral analysis at 3, 7 and 14 days post injury. We found a significant reduction in motor deficits following eB3-Fc infusion (91.7±7.7% of baseline control, *n*=12) compared with Fc controls (68.6±3.6% of baseline control, *n*=7) at 3 days after CCI injury (Figure 5e). Furthermore, sham mice infused with eB3-Fc had a similar motor response as sham animals infused with Fc molecules only, demonstrating that this effect is specific to CCI-induced deficits. Overall, these findings demonstrate that restoring ephrinB3 levels in the cortex and maintaining interactions with the EphB3 receptors can reduce cell death and limit motor deficits produced by moderate CCI injury.

### EphrinB3 infusion decreases neuronal cell death in the cortex following CCI injury

**Figure 6:**
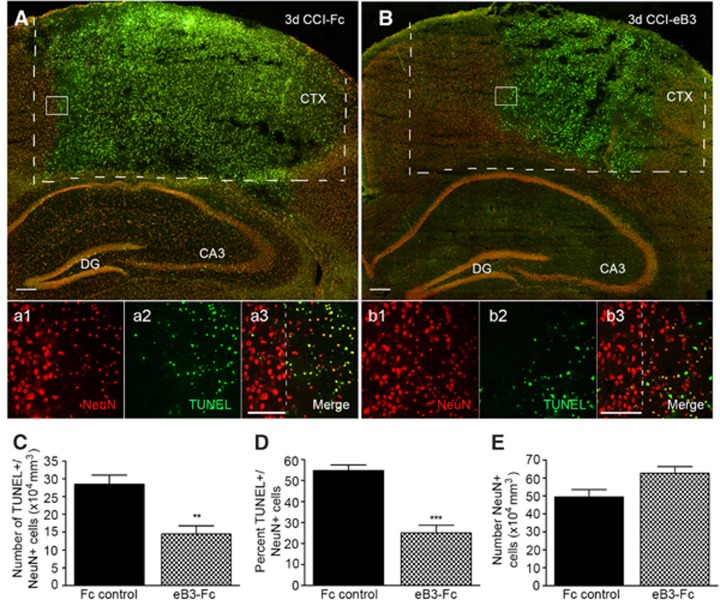
EphrinB3 infusion decreases neuronal tissue loss in the cortex following CCI injury. (**A** and **B**) Neuronal cell death was quantified following Fc and eB3-Fc infusion, respectively, by double-labeling for NeuN and TUNEL staining. (**A**) TUNEL-positive cells (green) were observed throughout the cortex, CA3 and dentate gyrus (DG). (a1-a3) High-magnification images of NeuN staining and TUNEL following 3 days Fc control infusion in wild-type mice. (**B**) Reduced TUNEL is observed in the cortex following eB3-Fc infusion compared with Fc controls. (b1-b3) High-magnification images of NeuN staining and TUNEL following eB3-Fc infusion. (**C**–**E**) Quantified data representing neuronal cell loss in the cortex following Fc control or eB3-Fc infusions. (**c**) The number of double-labeled TUNEL+/NeuN+ cells was significantly reduced in mice receiving eB3-Fc infusions compared with Fc controls. (**D**) The percentage (%) of total NeuN+ cells that also co-labeled with TUNEL staining was significantly reduced in mice receiving eB3-Fc infusions compared with Fc controls. (**E**) A non-significant increase in NeuN-positive cells was observed following eB3-Fc infusion compared with Fc control. ***P<0.001 and **P<0.01 compared with Fc controls. A and B scale bar=500 *μ*m. A3 and B3 scale bar=100 *μ*m

To further evaluate whether ephrinB3 infusion was neuroprotective in the cortex, we analyzed the amount of TUNEL-positive cells that co-labeled with the neuron-specific nuclear protein marker NeuN at 3 days following CCI injury. Serial coronal sections from CCI-injured mice infused with either Fc molecules (Figure 6a) or eB3-Fc (Figure 6b) were used and stained for TUNEL- and NeuN-positive cells. Visible reductions in TUNEL labeling and infarct volume were observed in the cortex of eB3-Fc infused mice compared with Fc controls. In addition, the total number of cells positive for both TUNEL and NeuN stainings was reduced in the cortex of eB3-Fc-infused mice (144 800±23 270) compared with Fc controls (284 800±25 870; Figure 6c). Furthermore, the percentage of TUNEL-positive neurons in the cortex at 3 days post CCI injury was significantly reduced in wild-type mice that received eB3-Fc infusion (25.1±3.6%) compared with Fc controls (54.7±2.7% Figure 6d). There were greater numbers of NeuN-positive cells in animals infused with eB3-Fc (576 800±20 580 per *μ*m^3^) as compared with Fc controls (493 800±41 130) but this difference was not significant (Figure 6e). These data suggest that application of soluble ephrinB3 can substantially limit the extent of neuronal cell death in the cortex at 3 days following CCI injury.

### EphB3 shows dependence receptor attributes

**Figure 7:**
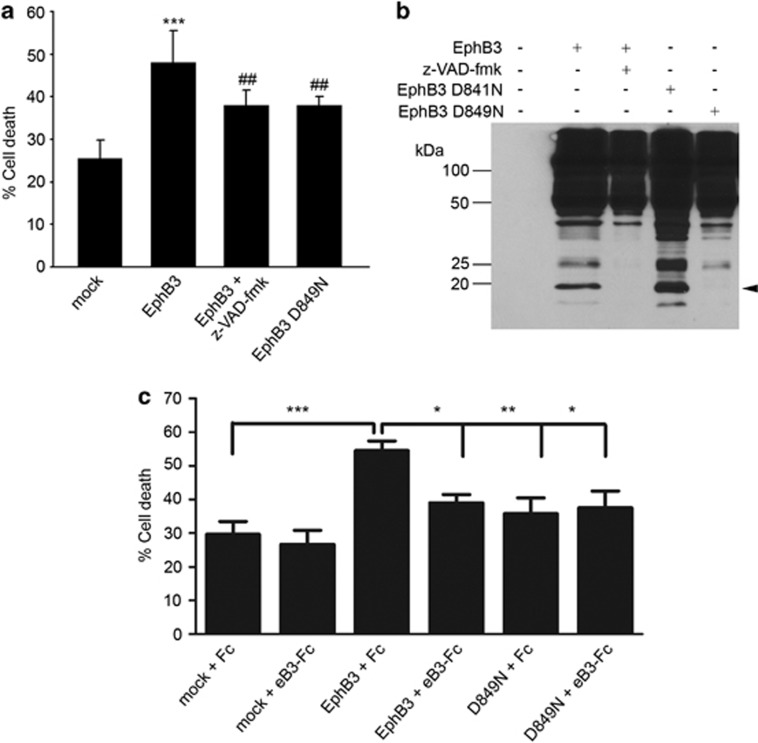
EphB3 overexpression in HEK293T and SY5Y cells leads to increased receptor cleavage and cell death. (**a**) HEK293T cell cultures overexpressing EphB3 showed greater cell death at 48 h than cells transfected with a mock vector as measured by Trypan blue exclusion. Addition of the caspase inhibitor, z-VAD-fmk or mutation of the aspartic acid residue in the position 849 to asparagine (D849N) showed a partial but significant reduction in cell death. (**b**) EphB3 was expressed in HEK293T cells in the absence of serum for 24 h to trigger cleavage of a 20-kDa fragment shown by western blot analysis. EphB3 cleavage was blocked in the presence of z-VAD-fmk and in EphB3 D849N mutants but not in EphB3 D841N mutants. (**c**) Cell death is enhanced by EphB3 overexpression in SY5Y cells at 48 h after transfection, which is blocked by application of 1 mg/ml eB3-Fc. Overexpression of the D849N mutant does not induce cell death. ***P<0.001 compared with Fc controls and ^##^*P*<0.01 compared with EphB3. ***P<0.001; **P<0.01; *P<0.05

Our *in vivo* data suggests that EphB3 may function as a dependence receptor to induce cell death in the cortex following TBI. A hallmark of many dependence receptors involves receptor modification through intracellular cleavage by caspase or caspase-like molecules, as previously shown with EphA4.^29^ To further examine the involvement of the EphB3 receptor in mediating caspase-dependent cell death, EphB3 was transiently overexpressed in human embryonic kidney (HEK)-293T cells to evaluate the cleavage response. Using trypan blue to assess cell death, we observed a significant increase in cell death following EphB3 overexpression in HEK293T cells as compared with mock vector controls (Figure 7a), supporting our *in vivo* observations. Western blot analysis following full-length EphB3 overexpression and serum deprivation revealed a lower migrating band at approximately 20 kDa (Figure 7b). Treatment with a broad range caspase inhibitor, z-VAD-fmk significantly blocked EphB3-induced cell death (Figure 7a) and EphB3 cleavage (Figure 7b), supporting a caspase-dependent mechanism. To further evaluate direct cleavage of the EphB3 intracellular domain by caspases, we modified the aspartic acid residues in regions that would lead to the generation of a 20-kDa fragment upon cleavage. Of the eight aspartic acid sites, we mutated (residues 753, 771, 780, 781, 815, 836, 841, 849), only the D>N mutation at residue 849 decreased cell death (Figure 7a). Similarly, the mutation D849N, but not D841N, prevented caspase-mediated cleavage (Figure 7b). Next, EphB3 and the EphB3 D849N mutant were transiently overexpressed in the human neuroblastoma cell line SY5Y in the presence and absence of eB3-Fc. We observed a significant increase in cell death following EphB3 overexpression in SY5Y cells as compared with mock vector controls (Figure 7c), which could be blocked by stimulating SY5Y cells with eB3-Fc. Similarly, this effect attenuated cell transfection with the D849N mutant with or without eB3-Fc. Together, these findings suggest that EphB3 may mediate neuronal cell death through a novel dependence receptor mechanism, which can be prevented with ephrinB3 treatment following brain trauma.

## Discussion

TBI initiates a complex cascade of events that leads to progressive injury and tissue loss. This study examines a novel mechanism of receptor-mediated cell death following CCI injury, where EphB3 functions as a pro-cell death dependence receptor. We find that EphB3 is present on MAP-2 expressing neurons, which closely associate with ephrinB3 ligand in the cortex and hippocampus. Astrocytes and oligodendrocytes have been shown to be major sources of ephrinB3 ligand.^31, 32^ Our studies *in vitro* and *in vivo* analysis suggest that neuronal-specific EphB3 may induce dependence receptor-mediated cell death following the reduced expression or interaction with ephrinB3 in the adult CCI-injured forebrain that leads to reduced motor performance, a phenotype that is reversed upon administration of ephrinB3-Fc or in the absence of EphB3 (EphB3^−/−^).

Motor deficits associated with CCI injury are likely the result of direct damage to motor cortex; however, subcortical regions are also damaged, such as CC and caudate-putamen, which may all contribute to motor dysfunction. Owing to the observation of ephrinB3 expression in deep cortical layers, our results support the possibility of it interacting with EphB3-expressing neurons in the motor cortex, although ephrinB3 is also highly expressed in both the CC and caudate-putamen. Finally, it is also possible that other supporting glia expressing EphB3 could contribute to the motor deficits observed following CCI injury. We do not believe our observations are the result of developmental influences, as motor defects are not observed in adult EphB3^−/−^ mice nor in sham-injured EphB3^−/−^ mice. This is further supported by our ability to reverse CCI-induced motor deficits following ephrinB3-Fc infusion. Like other dependence receptors, EphB3 acts as a caspase substrate and undergoes C-terminal cleavage that produces a 20-kDa fragment and ultimately leads to cell death. We cannot rule out that application of saturating amounts of ephrinB3-Fc to block cell death may not only compensate for the downregulation of ephrinB3 in the cortex but also disruption of membrane-bound ephrinB3 interactions with EphB3 in both cortical and subcortical tissues. Alternatively, reduced TUNEL staining and increased neuronal survival in the cortex supports the involvement of EphB3-mediated cell death in the motor cortex. In the future, it will be important to demonstrate Eph receptor cleavage in injured cells early before apoptotic cell loss; however, this has proven difficult to ascertain for several reasons. First, detection of a ∼20-kDa EphB3 C-terminal fragment requires a highly specific antibody, which is currently not available and has been difficult to develop. In fact, Eph receptors are highly conserved and generating a site-specific antibody is extremely challenging. Second, receptor cleavage is an early injury event that leads to cell death, so at any given time the populations that contain this cleavage product are eliminated. Thus, in order to address *in vivo* cleavage within the complex TBI environment, we will need to take a transgenic approach to develop cell-specific transgenic overexpressers with epitope tag and/or transgenic mice containing the D849N ‘non-cleavable' mutation. Overall, these studies describe a novel mechanism of cell death following TBI as well as a potential therapeutic strategy to reduce injury onset and progression.

Dependence receptors depend on their ligand to have supportive roles during development or homeostasis, but can convert to a pro-apoptotic receptor in the absence of ligand stimulation.^33, 34, 35, 36, 37^ A recent report by del Rio and colleagues identified a unique dependence-associated receptor transmembrane motif that is common to many described dependence receptors.^38^ This dependence-associated receptor transmembrane motif is present on both EphB3 and EphA4. Although EphB3 has a high homology with both EphB1 and EphB2, EphA4 is the only other Eph receptor currently shown to function as a dependence receptor.^29^ EphA4^29^ and EphB3 (Figure 7) have a single intracellular cleavage site (residues 773/774 and 849, respectively) that are critical for caspase cleavage. These two receptors are co-expressed in many cell types, including cortical neurons,^31^ suggesting they may have redundant roles, although this is clearly not the case for many cell functions, as EphB3 and EphA4 knockout mice are not phenotypically similar. This diversity between highly homologous receptors that potentially interact with common ligands may result from higher order receptor complexes, interactions with membrane proteins or unique intracellular signaling intermediates.^39^ Functional diversity may also exist for an individual receptor; for example, EphB3 has been demonstrated to have opposing effects on non-small-cell lung cancer metastasis. Several studies have shown that EphB3 can both promote tumor metastasis^40^ and suppress metastatic progression.^41^ The authors attribute these pro- and anti-apoptotic properties to its ligand interaction and kinase activity, although the mechanisms of action remain poorly defined. It is clear that a better understanding of how Eph interactions regulate cell survival following CNS injury is warranted.

EphB3 has been shown to have a critical role in cell expansion and cell death in adult neurogenesis.^30^ EphB3 limits neural stem/progenitor cell proliferation in the adult subventricular zone thereby maintaining homeostasis of this highly proliferative neurogenic region.^29, 30^ Here, we show that EphB3 may also have deleterious roles on residential cells in the adult cortex after traumatic injury. The most convincing evidence that EphB3 functions as a dependence receptor following CCI injury comes from analysis of the ephrinB3^−/−^ and EphB3^−/−^ mice, where deficiencies in the ligand–receptor partners are not phenocopied. However, we would have anticipated increased cell death in the cortex of ephrinB3^−/−^ mice compared with wild type after CCI injury but the level of damage appeared consistently the same. Previously, we demonstrated the possibility of EphB3 functioning as a dependence receptor by showing opposite effects on cell death between ephrinB3^−/−^ and EphB3^−/−^ mice in the naïve SVZ, an area that lies directly adjacent to heavy ephrinB3-expressing regions of the CC and striatum and whose levels of ephrinB3 remain unchanged after CCI injury.^30^ On the other hand, the cortex expresses much lower levels of ephrinB3, where in layers I–III it is virtually absent. It is possible that downregulation of already low levels of ephrinB3 and dissociation of existing EphB3/ephrinB3 interactions through extensive cellular disruption and tissue loss in the cortex of wild-type mice may mimic an injured ephrinB3^−/−^ environment and therefore result in a similar outcome. Based on that rationale, we could therefore expect greater differences to occur in the CC between our knockout mice due to higher levels of ephrinB3 expression. Future studies that focus on oligodendrocyte or oligodendrocyte precursor functions in this region may be warranted.

There are a number of membrane receptors that mediate cell death after CNS injury, the most notable being the tumor necrosis factor (TNF) superfamily.^42^ Similar to the Eph family, the TNF receptor superfamily has an extracellular ligand-binding domain, a transmembrane domain and a C-terminal domain containing a death domain that is critical for induction of cell death. Unlike dependence receptors, TNF receptor family members, such as Fas, TNF-receptor 1, TRAIL-R1 and TRAIL-R2 require ligand activation and do not require cleavage of the C-terminal tail to expose the death domain. Thus, TNF receptor-induced cell death results from an active release of TNF, whereas Eph-mediated dependence receptor cell death requires multifunctional responses that include ligand–receptor disruption and receptor modification. Together, these diverse cell death signals contribute to the tissue damage following TBI.

## Materials and Methods

### Animals

The generation of the mutant CD1 mice and genotyping using PCR analysis has been previously described.^43, 44, 45, 46^ Animals were killed by decapitation under anesthesia; the brain was immediately removed and frozen in Tissue-Tek OCT (Sakura, Torrance, CA, USA) then preserved at −80 °C until further processing. Procedures related to animal use and care was approved by the University of Miami Animal Use and Care Committee.

### CCI and infusion

Male mice ages 2–4 months were anesthetized with ketamine and xylazine by i.p. injection and positioned in a stereotaxic frame. Body temperature was monitored with a rectal probe and maintained at 37 °C with a controlled heating pad set. A 5-mm craniotomy was made using a portable drill over the right parieto-temporal cortex (−2.0 mm A/P and 2.0 mm lateral from bregma). Injury was induced by moderate CCI using the eCCI-6.3 device (Custom Design and Fabrication, Richmond, VA, USA) at a velocity of 6 m/s, depth of 0.5 mm and 150 ms impact duration. Sham controls received craniotomy only. Following injury, the incision was closed using Vetbond tissue adhesive (3M, St. Paul, MN, USA) and the animals were placed into a heated cage to maintain body temperature for 1 h post injury. EphrinB3-Fc or control human Fc molecules (R & D Systems, Minneapolis, MN, USA) at a concentration of 140 *μ*g/ml were preclustered with goat anti-human Fc antibodies (ratio 1 : 5; Jackson Research Laboratories, Inc, West Grove, PA, USA) in phosphate-buffered saline (PBS) for 2 h. Alzet osmotic pumps (Alzet Durect Corp, Cupertino, CA, USA) were preloaded with clustered ephrinB3-Fc or Fc controls, placed in lateral ventricle (from bregma: A/P −0.5 mm; lateral 0.7 mm) using stereotactic holder, and secured to cranium with glue. Pumps were placed under the skin of dorsal neck region for an infusion over a 3-day period (12 *μ*l total volume dispensed per day at 0.5 *μ*l/hr rate).

### Lesion and hemispheric volumes

Five 30 *μ*m sections from bregma levels −1.3 to −2.2 mm, 300 *μ*m apart, were stained with cresyl violet for Nissl substance and digitally photographed using an Olympus BX51TRF microscope (Olympus America, Center Valley, PA, USA) with a × 4 objective. The peripheries of the contralateral and ipsilateral hemispheres were traced on each section by an evaluator blinded to the injury and treatment status of each animal, and the cortical lesion volume was calculated using a calibrated image analysis routine, using the software Neurolucida (National Institutes of Health, Bethesda, MD, USA). Lesion volumes were quantified by tracing the contralateral cortex, and projecting the inverted contour over the ipsilateral cortex. The lesion contour was demarcated by pyknotic neurons, hemorrhage and edema. Contusion areas were calculated for five coronal levels at or around the lesion site (−1.3, −1.6, −1.9, −2.2 mm posterior from bregma).

### Stereology

Fresh frozen coronal, 30-*μ*m-thick cryostat brain serial sections were collected from −1.3 to −2.2 bregma (total thickness of the examined tissue was 1500 *μ*m). Cell death was assessed using TdT-mediated dUTP nick-end labeling (TUNEL) staining kit as previously described^30^ with double-labeling for NeuN to identify neurons. The total number of NeuN- and TUNEL-positive cells within 1500 μm of cortical tissue were analyzed using a motorized Olympus BX51TRF microscope, Optronix cooled video camera and MicroBrightField StereoInvestigator software package (MBF Bioscience, Williston, VT, USA). To perform nonbiased cell number estimation, the optical fractionator method and optical dissector probe were used. The cortical lesion, CA3 and dentate gyrus regions were overlaid with contours that covered the entire region of interest using a × 10 magnification stained with Nissl to visualize/identify the anatomical structures in every fifth tissue section (30 *μ*m). Next, a grid of 100 × 100 *μ*m^2^ was placed over this area, and the number of TUNEL-, NeuN- and NeuN/TUNEL-positive cells were randomly counted using optical fractionator at × 63 magnification (sampling box: 50 × 50 *μ*m^2^).

### Staining procedures

For immunostaining, fresh frozen tissue sections were fixed for 5 min with 10% buffered formalin, washed three times with 1 × PBS, permeabilized for 10 min in 2 : 1 ethanol/acetic acid solution, washed three times with PBS and permeabilized with 0.4% Triton X-100 followed by TUNEL labeling. DeadEnd fluorometric TUNEL kit staining was carried out according to the manufacturer's instructions (Promega, Madison, WI, USA). For double labeling with anti-NeuN, the sections were post-fixed with 10% formalin, subsequently rinsed three times with 1 × PBS and blocked in 5% BSA/0.2% Triton in PBS for 1 h, then mouse monoclonal anti-NeuN (1/200 in block, Millipore, Cambridge, UK) was applied overnight at 4 °C. Sections were washed three times with 1 × PBS and AlexaFluor 594-conjugated secondary antibodies (Molecular Probes, Carlsbad, CA, USA) were applied for 1 h at room temperature. Sections were finally washed with 1 × PBS, counterstained with Hoechst (0.1 *μ*g/ml, Molecular Probes) and mounted in Pro-Long anti-fade mounting solution (Molecular probes). Expression studies used anti-EphB3 (1/200, Abcam, Cambridge, UK), anti- microtubule-associated protein 2 (1/1000, Millipore) and anti-GFAP (1/1000, Dako, Cambridge, UK) antibodies.

### RotaRod assessment

Motor function was tested between 3 and 14 days after injury by observers unaware of experimental groups using a RotaRod for mice. The initial velocity was 4 r.p.m. and accelerated to 60 r.p.m. over 10 min. Animals were trained for three consecutive days before CCI injury with four trials (2 min resting in between) each day. Each trial ended when the animal fell off the RotaRod or gripped the rod and passively spun more than once. A baseline was collected on the last day of training. Evaluation of motor function after injury was based on individual scores relative to their baseline latencies.

### Cell culture

SY5Y human neuroblastoma cell line, generously donated by Dr. Marion Ehrich VMRCVM, were maintained in L15 media containing 10% FBS. For transient transfection, 60 000 cells were plated per well in a 24-well plate a day before transfection. A complete media change was performed 30 min before transfection, during which time the plasmids pcDNA3.1-V5, pcDNA3.1-V5-EphB3 and pcDNA3.1-V5-EphB3D849N were mixed with Truefect United Biosystems (Herndon, VA, USA) in DMEM/high glucose (Gibco, Langley, OK, USA) according to manufacturer's recommendation and then 25 *μ*l of each complex was added to each respective wells and incubated for 5 h. Media was then removed, washed once with DMEM/high glucose and 500 *μ*l of DMEM/high glucose containing either pre-clusted Fc (0.5 *μ*g/ml) or ephrinB3-Fc (1.0 *μ*g/ml) were added. Forty-eight hours after transfection, trypan blue assay was performed to assess the percentage of cell death for each condition.

### Western blot analysis

Proteins from cortical tissue (carefully dissociated from the CC) were extracted by lysis in RIPA buffer (pH 7.5, 1% NP-40, 1% sodium-deoxycholate, 0.1% SDS, 0.15 M NaCl, 2 mM EDTA and 0.01 M sodium phosphate) in the presence of Complete protease inhibitor cocktail (Roche, Florence, SC, USA) and phosphatase inhibitor cocktail 2 (Sigma-Aldrich, St. Louis, MO, USA). Supernatant was collected by centrifuging at 14 000 × *g* for 30 min at 4 °C and the Lowry assay was used for the determination of protein concentration (Pierce, Rockford, IL, USA). Protein samples were resolved on 10% SDS-PAGE gels and blotted onto PVDF membranes that were blocked with 5% BSA or milk in TBST buffer (20 mM Tris, 137 mM NaCl, 0.1% Tween) and incubated with primary antibodies against ephrinB3 (1 : 200, Invitrogen, Carlsbad, CA, USA), EphB3 (1 : 8000, Abcam), phospho-tyrosine (1 : 1000, Cell Signaling, Danvers, MA, USA) or *β*-actin (1 : 8000, Cell Signaling) diluted in TBST-3% BSA or milk overnight at 4 °C. HRP-conjugated secondary antibodies (Jackson Research Laboratories, Inc) in blocking solution were applied to the membrane after four TBST washes and incubated for 2 h at room temperature. Blots were quantified by densitometry using acquisition into Adobe Photoshop and analyzed by the NIH Image software (National Institutes of Health). The level of protein expression were normalized to *β*-actin then represented as percentage of sham-injured control levels for each time point.

### Statistical analysis

Data were graphed using GraphPad Prism, version 4 (GraphPad Software, Inc., San Diego, CA, USA). Student's two-tailed *t*-test was used for comparison of two experimental groups. Multiple comparisons were done using one-way ANOVA followed by Tukey test for multiple pairwise examinations. Changes were identified as significant if *P* was less than 0.05. Mean values were reported together with the standard error of mean (S.E.M.)

## Abbreviations

CCI: controlled cortical impact
TUNEL: terminal deoxynucleotidyl transferase-dUTP nick end labeling
eB3-Fc: ephrinB3-Fc molecule
CC: corpus callosum
HEK: human embryonic kidney
CNS: central nervous system
TBI: traumatic brain injury
